# Effects of a Post-Shock Injection of the Kappa Opioid Receptor Antagonist Norbinaltorphimine (norBNI) on Fear and Anxiety in Rats

**DOI:** 10.1371/journal.pone.0049669

**Published:** 2012-11-14

**Authors:** Benjamin Rogala, Yonghui Li, Sa Li, Xiaoyu Chen, Gilbert J. Kirouac

**Affiliations:** 1 Department of Oral Biology, Faculty of Dentistry, University of Manitoba, Winnipeg, Manitoba, Canada; 2 Key Laboratory of Mental Health, Institute of Psychology, Chinese Academy of Sciences, Beijing, China; 3 Department of Psychiatry, Faculty of Medicine, University of Manitoba, Winnipeg, Manitoba, Canada; University of Regensburg, Germany

## Abstract

Exposure of rats to footshocks leads to an enduring behavioral state involving generalized fear responses and avoidance. Recent evidence suggests that the expression of negative emotional behaviors produced by a stressor is in part mediated by dynorphin and its main receptor, the kappa opioid receptor (KOR). The purpose of this study was to determine if a subcutaneous injection of the long-acting KOR antagonist norbinaltorphimine (norBNI; 15.0 and 30.0 mg/kg) given 2 days after an acute exposure of rats to footshooks (5×2 s episodes of 1.5 mA delivered over 5 min) attenuates the expression of lasting fear and anxiety. We report that exposure of rats to acute footshock produced long-lasting (>4 weeks) fear (freezing) and anxiety (avoidance of an open area in the defensive withdrawal test). The 30 mg dose of norBNI attenuated the fear expressed when shock rats were placed in the shock context at Day 9 but not Day 27 post-shock. The same dose of norBNI had no effect on the expression of generalized fear produced when shock rats were placed in a novel chamber at Days 8 and 24. In contrast, the 30 mg dose of norBNI produced consistent anxiolytic effects in shock and nonshock rats. First, the 30 mg dose was found to decrease the latency to enter the open field in the defensive withdrawal test done 30 days after the shock exposure. Second, the same high dose also had anxiolytic effects in both nonshock and shock rats as evidence by a decrease in the mean time spent in the withdrawal box. The present study shows that systemic injection of the KOR antagonist norBNI had mixed effect on fear. In contrast, norBNI had an anxiolytic effect which included the attenuation of the enhanced avoidance of a novel area produced by a prior shock experience.

## Introduction

Clinical evidence indicates that many individuals exposed to a severe trauma involving intense fear subsequently exhibit a strong emotional reaction when confronted with reminders of the trauma situation [1]–[2]. In addition, these individuals often display fear or anxiety in situations that would not normally elicit this reaction [1]–[2]. The generalization of fear to situations not directly related to the trauma can lead to anxiety and avoidance of normal day-to-day situations [3] which may lead to the diagnosis of post-traumatic stress disorder (PTSD) in individuals in which the symptoms last longer than one month [4]. The discovery of more effective treatments is essential since PTSD symptoms may last for years to decades in some individuals despite these people having received psychological and pharmacological treatment [5]–[6].

Similar to the clinical situation, rodents exposed to a single episode of moderately intense footshocks (1.5 to 2.0 mA) not only show a strong fear response when re-exposed to the shock apparatus associated with the shock experience but also display an increase of fear-like (immobility) response when exposed to novel environment or loud noises [7]–[14]. A number of studies have also shown that rodents pre-exposed to electrical shock show enhanced avoidance or anxiety in situations involving novel conspecific, objects, or test areas [8], [10], [14]–[17]. The anxiety displayed by rodents previously exposed to shock results from an adaptive response where potentially fearful situations are avoided or approached with caution. As shown recently, generalized fear and learning mechanisms appear to contribute to avoidance of fear-inducing situations [18].

There has been a surge of recent interest in the possibility that the kappa opioid receptor (KOR) and prodynorphin derived peptides (dynorphins), which act with high specificity at KORs [19]–[20], modulate negative emotional states following exposure to stress [21]–[23]. Indeed, there are a number of studies showing that systemic and central injections of KOR agonists produce dysphoria, anxiety and pro-depressive states in humans and rodents [24]–[28] while KOR antagonists attenuate the anxiety- and depression-like behaviors [29]–[33]. There is also ample evidence showing that disrupting the synthesis of prodynorphin derived peptides or blocking KOR with antagonists reduces the negative emotional states associated with a previous exposure of rodents to forced swimming, social defeat, and footshock stress [26], [34]–[37] and has anxiolytic effects in non-stressed rodents [29]–[30], [33]. In addition, a role for KOR in fear conditioning has been provided by studies showing that central administration of a KOR antagonist interfered with freezing and fear-potentiated startle [29], [38]. Taken together, these findings suggest that blocking KORs before or at the time of the stress episode can attenuate negative emotional behaviors expressed 1 to 2 days after exposure to a stressful situation [26], [34]–[37]. In contrast, no studies have reported that blocking of KORs after a stress episode is effective in reducing the negative emotional behaviors that result from the stressor.

As described above, exposure of rats to a brief episode of relatively intense footshocks produces long-lasting fear/anxiety and provides a useful model to examine if a KOR antagonist could have therapeutic effects if given to people after a trauma experience. The present study was done to determine if a single post-shock injection of the long-lasting antagonist norbinaltorphimine (norBNI) prevents the expression of the fear- and anxiety-like behaviors over a 4 week period in rats. This is of special interests because norBNI and most of the more specific KOR antagonists have slow onset latencies and maintain their pharmacological effects for weeks following a single administration [39]–[40].

## Materials and Methods

### Ethics Statement

The experimental procedures were in compliance with the Canadian Council on Animal Care and the experimental protocol was approved by Research Ethics Review Board of the University of Manitoba (protocol number 09-057) and every effort was made to minimize the suffering of the animals.

### Animals and Housing

Six week old male Sprague-Dawley rats (University of Manitoba, Canada) weighing 140–150 g at the time of first arrival to the animal facility were pair-housed in plastic cages in a colony room on a 12 h/12 h light/dark cycle (lights on 06:00) with controlled temperature (20–24°C) and humidity (40–70%). All rats had free access to food and water in their home cages and were handled for 2 min on alternate days during a 10-day adaptation period. All the behavioral tests were done in the light cycle of the day (09:00–17:00) in a different room from the colony room. Testing during the light phase was done to enhance the expression of fear and anxiety following footshock exposure.

### Experimental Design

The parameters for the shock procedure as well as the timing of the behavioral tests were according to work previously described from our laboratory [14] which was based on methods used by a number of laboratories [7]–[13]. At 8 weeks of age, rats were randomly assigned to different experimental conditions as follow. On Day 0, rats were transferred to the testing room where some of the rats received a single episode of footshocks (n = 29) while the remainder of the animals were placed in the chamber without receiving footshocks (n = 26). We have previously shown that rats show individual differences in their reaction to the footshock exposure [14]. Accordingly, on Day 1, rats were placed in a small open field to determine the amount of generalized fear expressed early after the footshock exposure and to generate homogeneous groups for the treatment conditions. On Day 2, subgroups of nonshock and shock rats received injections of norBNI at 15.0 mg/kg (n = 8 and 11 for nonshock and shock, respectively), 30.0 mg/kg (n = 9 and 9 for nonshock and shock, respectively) or vehicle (n = 9 and 9 for nonshock and shock, respectively). A series of behavioral tests were done at early (Days 8–9) and later (Days 24–30) time points to measure fear- and avoidance-like behaviors and to determine the effect of norBNI on these measures (Table 1; each rat was exposed to all tests). The order and time of the behavioral tests were counterbalanced among the different drug treatment groups. The novel test chambers, open field, and defensive withdrawal chambers were cleaned with liquinox (0.5%) whereas the shock chamber was cleaned with ethanol (10.0%) after each rat exposure. Analysis of behavioral tests was done by experimenters blind to the treatment conditions.

**Table 1:** Timeline of the behavioral tests.

Day 0	Shock chamber (shock/nonshock)
Day 1	Small open field test
Day 2	Treatment with saline or norBNI
Day 8	Novel square chamber
Day 9	Shock chamber exposure (no shock)
Day 24	Large open field test
Day 27	Shock chamber exposure (no shock)
Day 30	Defensive withdrawal test

### Generation of Homogenous Shock and Drug Injection Groups

We have shown previously that shock rats display a range of post-shock fear generalization and that rats showing a higher level of fear generalization exhibit more anxiety 4 weeks after shock exposure [14]. To generate homogenous treatment groups of shock rats, post-shock fear generalization was assessed on Day 1 by measuring the amount of freezing expressed in rats placed in a small open field (made of black Plexiglas and measuring L65 cm×W40 cm×H50 cm) for 3 min. Freezing, defined as complete lack of body movement except breathing movements [41], was expressed as the percentage of time the rats spent freezing during the test period. The range of percentage of time spent freezing for nonshock rats was 0 to 2.6% and these rats were randomly assigned to treatment groups. The range of percentage of the time spent freezing for shock rats was 0 to 48.3% and based on these responses, shock rats were assigned to form 3 treatment groups with similar means (saline = 23.33±6.37%; 15 mg = 19.70±3.94%; 30 mg = 24.04±3.98). The KOR antagonist norBNI (Tocris, U.K) was dissolved in sterile saline (15.0 and 30.0 mg/ml; with the high dose dissolved by gentle warming in a 60°C water bath) on the same day as the injections. Treated rats received norBNI injections (15.0 and 30.0 mg/kg, s.c.) in a volume of 1.0 ml/kg body weight on Day 2 following footshock exposure. The antagonist norBNI has slow onset latency and maintains pharmacological effects for weeks [39]–[40]. The 15.0 and 30.0 mg/kg doses used in the present study are similar to what have been previously reported to be effective for antidepressive and anxiolytic effects in rodents [26], [29], [31], [34]–[35], [37]. While norBNI has a short lasting antagonist effect on mu opioid receptor (2–4 hours), the long-lasting antagonist effect exists only for the KORs [39], [42].

### Behavioral Procedures

Rats were transferred individually to the behavioral testing room and placed into a shock chamber with a grid floor (MED Associates, St. Albans, VT, USA). After a 2 min acclimation period, rats were exposed to footshocks (5×2 s of 1.5 mA shock with an inter-shock period of 10–50 s presented randomly over 3 min) administrated by a commercially available scrambled stimulator (MED Associates). The rats were kept in the chamber for another 60 s before they were returned to their home cages. The shock chamber was cleaned with ethanol and the bedding under the grid floor was changed for each animal. The rats in the nonshock group were exposed to the shock chamber in the same way except that the stimulator was turned off. Rats were transported and shocked one at a time so that other rats would not be exposed to auditory and olfactory responses produced during the shock procedure.

The post-shock generalized fear response was assessed on Day 8 by placing rats in a clear plastic chamber (L22 cm×W28 cm×H35 cm) for 5 min. The activity of the rats was recorded using a video camera and all of the video was subsequently scored for presence of freezing by two experimenters blind to the experimental conditions. The reliability score between raters ranged between 0.94–0.97 (correlation coefficient) and the data from two observers were averaged for statistical analysis.

Fear generalization was also examined on Day 24 by placing rats in the center area of a large open field (L80 cm×W80 cm×H40 cm) made of black Plexiglass for 5 min. The chamber was illuminated with a light of 8–10 lux. The conditioned fear response was measured on Days 9 and 27 after the footshock exposure by placing the rats in the shock chamber for 5 min (no shock delivered). The behavioral activity in the shock chamber was recorded and analyzed as described above.

The defensive withdrawal test was done as originally described [43] on Day 30 to measure avoidance to novel environment using an apparatus that consisted of an open field (L80 cm×W80 cm×H40 cm with a black floor and green opaque walls) and a smaller movable black box (L25 cm×W20 cm×H15 cm made of black opaque plastic walls and floor) with a sliding door. The movable withdrawal box was placed 20 cm away from one of the corners of the open field. The light level in the test chamber was approximately 7 lux and the test protocol was similar to what was described in previous studies [44]–[45]. The rat was placed in the withdrawal box which was then placed into the open field with the door facing one corner. Two minutes later, the sliding door was removed to allow the rat to freely explore the open field. The behaviors of the rat in the open field were recorded for 10 min using a video camera. The latency to enter the open field (four paws into the open arena) and the time spent in the withdrawal box (total time spent in the withdrawal box/number of entries) were quantified.

### Statistical Analysis

The study was a 2 (shock vs nonshock) ×3 (saline, 15, and 30 mg/kg) factor design. Behavioral test day (early vs. late) was not analyzed as factors and separate statistical tests were done for those data sets. The between factor data were analyzed using two-way ANOVA for main effects involving “shock” (nonshock and shock) and “norBNI” treatment (saline, 15 mg and 30 mg) as well as interaction effects between “shock” and “norBNI”. When a main effect or interaction was indicated by the two-way ANOVA, the within factor data (shock vs. nonshock) was further probed using one-way ANOVA to determine if norBNI had an effect on the behaviors examined. Least significant differences (LSD) post-hoc tests were used to identify which of the drug treatments produced the significant differences indicated by the one-way ANOVA. The statistical analysis was done using SPSS 19 and all data are shown as mean ± SEM.

## Results

### Generalized Fear

**Figure 1:**
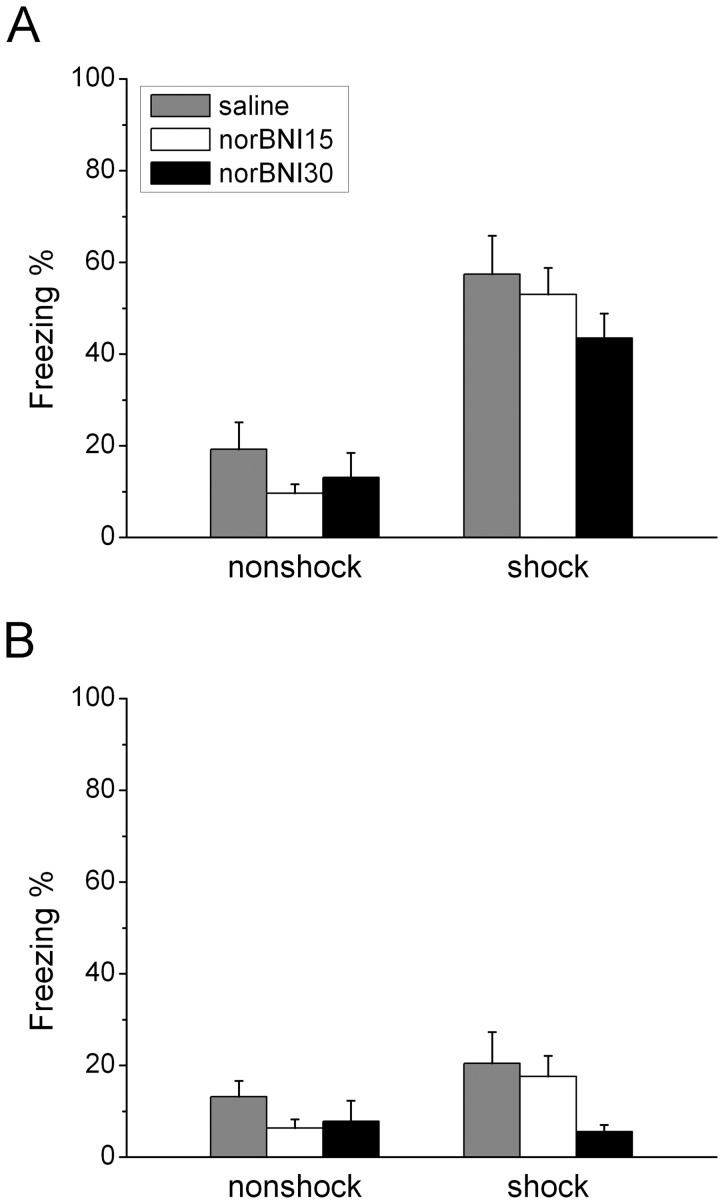
Effects of systemic injections of norbinaltorphimine (norBNI) on the generalized fear response on Day 8 (A) and Day 24 (B). The values represent mean ± SEM for this and all subsequent figures.

Animals exposed to an intense stressor involving fear will show a fear response when confronted with novel situations that are not directly related to the trauma experience [46]. In this study, fear generalization was assessed by measuring the amount of freezing expressed when rats were placed in a novel environment on Days 8 and 24. The two-way ANOVA revealed that shock rats showed more freezing on Day 8 (Fig. 1A; F_1,49_ = 59.653, *p*<0.001) but not on Day 24. There was no significant effect of “norBNI” on freezing on Days 8 or 24, nor was there an interaction effect between “shock” and “norBNI” on Days 8 or Day 24. In summary, shock rats showed more freezing when placed in a novel chamber on Day 8 and inactivation of KORs with norBNI did not affect the amount of freezing expressed.

### Conditioned Fear Response

**Figure 2:**
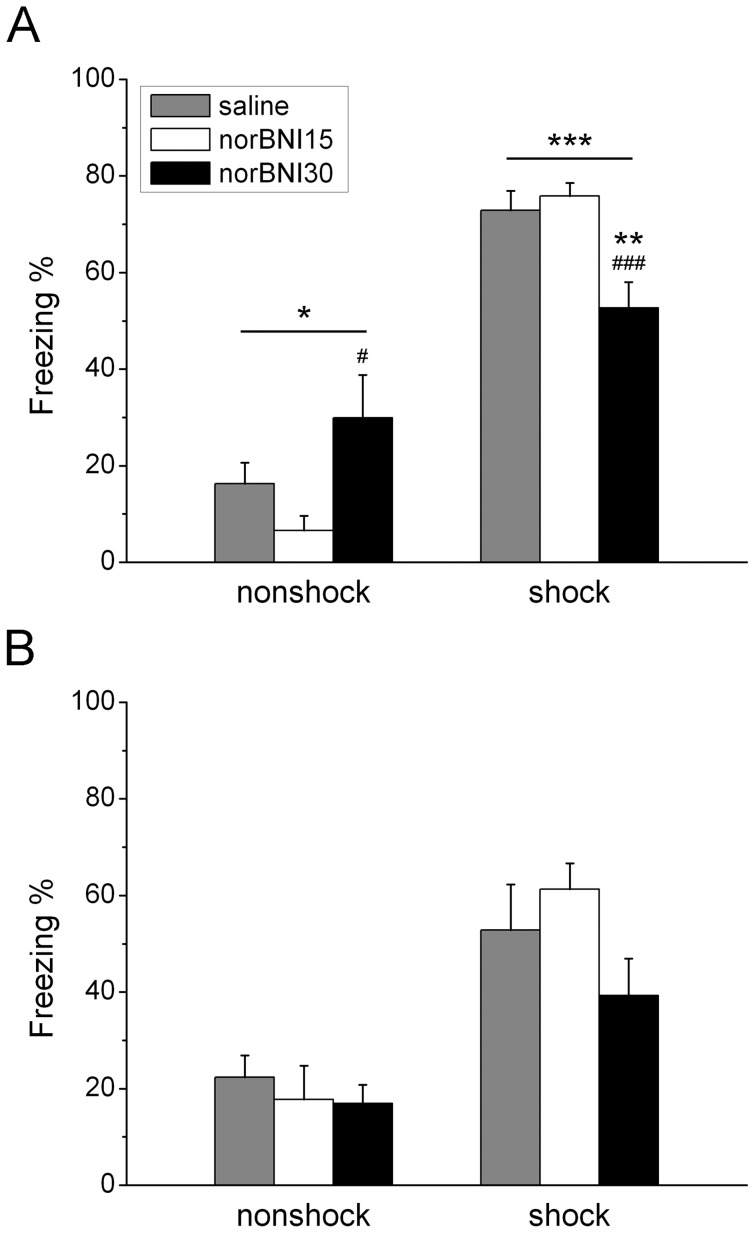
Effects of systemic injections of norbinaltorphimine (norBNI) on the conditioned fear response on Day 9 (A) and Day 27 (B). * indicates *p*<0.05, ** *p*<0.01, *** *p*<0.001 when comparisons are within groups or when compared to saline group; # indicates *p*<0.05, ### indicates *p*<0.001 when compared to the 15 mg/kg norBNI group.

The amount of freezing expressed when rats are re-exposed to the shock chamber was used to assess the strength of the conditioned fear response. The two-way ANOVA showed that shock rats expressed more freezing on Day 9 (Fig. 2A; F_1,49_ = 142.001, *p*<0.001) and Day 27 (Fig. 2B; F_1,49_ = 36.044, *p*<0.001). For test done on Day 9, there was an interaction effect between “norBNI” and “shock” (F_2,49_ = 11.137, *p*<0.001). The one-way ANOVA showed that norBNI had an effect on freezing duration in shock rat (F_2,26_ = 9.819, *p*<0.001) and nonshock rats (F_2,23_ = 3.564, *p*<0.05). The post-hoc analysis revealed that the 30.0 mg dose of norBNI significantly decreased freezing duration when compare to the 15.0 mg dose (*p*<0.001) and saline (*p*<0.01) in shock rats on Day 9. In contrast, the high dose of norBNI increased freezing in nonshock rats when compare to the low dose of norBNI (*p*<0.05) but not saline treated rats. There was no interaction effect between “norBNI” and “shock” for test done on Day 27. The results indicate that the high dose of norBNI attenuated the expression of conditioned fear in shock rats while increasing fear in nonshock rats when tested at an early time point.

### Avoidance of Open Spaces

**Figure 3:**
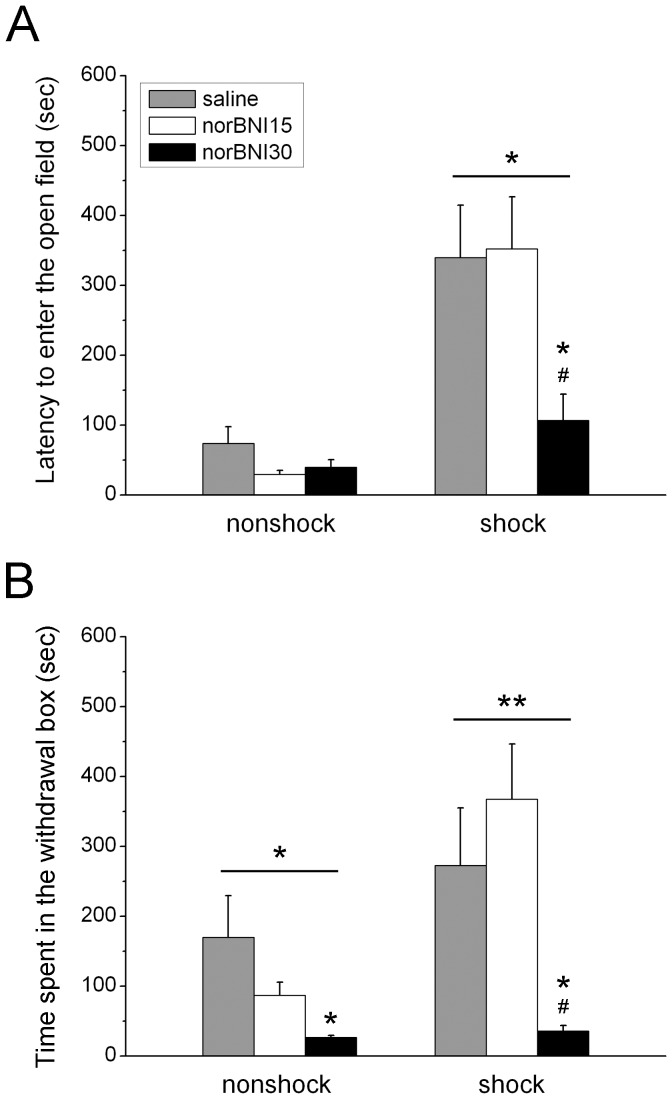
Effects of systemic injections of norbinaltorphimine (norBNI) on the latency to leave the withdrawal box (A) and the time spent in the withdrawal box (B) in the defensive withdrawal test. * indicates *p*<0.05, ** *p*<0.01 when comparisons are within groups or when compared to saline group; # indicates *p*<0.05, ## indicates *p*<0.01 when compared to the 15 mg/kg norBNI group.

The defensive withdrawal test was done to determine the effect of norBNI on the anxiety produced by footshock exposure. The two-way ANOVA demonstrated that shock rats had a higher latency to enter the open field (Fig. 3A; F_1,49_ = 27.399, *p*<0.001). Furthermore, the two-way ANOVA also indicated that there was a main effect for “norBNI” (Fig. 3A; F_2,49_ = 4.045, *p*<0.05) and an interaction effect between “norBNI” and “shock” (F_2,49_ = 3.463, *p*<0.05) on latency. The one-way analysis showed that there was an effect for norBNI on shock (F_2,26_ = 4.134, *p*<0.05) but not nonshock rats. Post-hoc analysis showed that the 30 mg dose of norBNI significantly decreased the latency when compare to the 15 mg dose (*p*<0.01) and the saline treated group (*p*<0.05). The two-way ANOVA indicated that there was a main effect for “shock” (Fig. 3B; F_2,49_ = 7.851, *p*<0.01) and “norBNI” (Fig. 3B; F_2,49_ = 7.536, *p*<0.001) on time spent in the withdrawal box. However, there was no interaction between “norBNI” and “shock”. The one-way ANOVA indicated that norBNI had significant effects on the time in both the nonshock (F_2,23_ = 3.828, *p*<0.05) and shock (F_2,26_ = 6.190, *p*<0.01) rats. For nonshock rats, the post-hoc analysis showed that the 30 mg dose significantly decreased the time in the withdrawal box when compared to the saline treated group (*p*<0.01). For the shock group, post-hoc analysis showed that the 30 mg dose significantly decreased the time when compare to the 15 mg dose (*p*<0.01) and the saline treated group (*p*<0.05). The results indicate that the 30 mg dose of norBNI decreased the latency for shock rats to leave the safety of the withdrawal box. In addition, the 30 mg dose decreased the time that nonshock and shock rats spent in the withdrawal box.

## Discussion

In this study, we investigated if inactivation of KORs with norBNI attenuated fear and anxiety in a rat model of PTSD. In general, acute exposure to footshocks (1.5 mA) produced long-lasting expression of fear- and anxiety-like behavioral responses (freezing and anxiety) in shock rats when they were placed in the same context where shock occurred or when exposed to novel environment. More importantly, the high dose of norBNI was effective in lowering the latency to leave the withdrawal box in shock rats indicating an anxiolytic effect of norBNI specific to shock rats. We found that the high dose of the KOR antagonist norBNI had anxiolytic effects in both nonshock and shock rats when we used the time spent in the withdrawal box as a measure of anxiety. We also report that norBNI slightly reduced the expression of conditioned fear 9 days after shock exposure. These results are consistent with other studies showing that treatment of rodents with a KOR antagonist before or immediately after (0.5 hr) a stress-related event interfered with some of the behavioral changes associated with the stress experience a few days later [26], [34]–[35], [37]. However, we show for the first time that a single post-shock injection of the KOR antagonist norBNI can attenuate anxiety produced in rats exposed to footshocks of 1.5 mA.

Evidence for a role for KOR in fear has been provided by a number of studies. First, central administration of norBNI prior to contextual fear conditioning was reported to interfere with the subsequent expression of freezing to the shock context [38]. Second, systemic injections of norBNI or JDTic, another KOR antagonist, prior to discriminative fear conditioning was shown to attenuate the expression of fear-potentiated startle responses [29]. However, the extended inactivation of KOR that results from the administration of these KOR antagonists makes it impossible to know from those studies if the drug treatment interfered with the acquisition or with the expression of the leaned fear response. In a third study, central administrations of norBNI after a discriminative fear conditioning procedure had no effect on subsequent expression of fear [47]. In contrast to this most recent study, we report in this paper that contextual fear expression at Day 9 was weakly attenuated in shock rats that had received a post-shock injection of 30 mg/kg norBNI. In addition, norBNI had inconsistent effects on fear produced when rats were exposed to novel or shock chamber. For example, the high dose of norBNI reduced contextual fear in shock rats at Day 9 while increasing fear in nonshock rats placed in the shock chamber. Second, norBNI did not significantly lower the level of generalized fear in shock rats given the fact that freezing to the novel chamber was nearly as high as that expressed in the shock chamber. Based on our experiments as well as previous studies, we conclude that the role of KOR in fear is complex and that norBNI treatment after a strong fear inducing episode may not be a useful approach for modulating fear expression.

Shock rats displayed more freezing when exposed to novel chambers, a behavioral response which is taken as a sign that fear which is normally associated with novelty is more easily aroused or that the experience of the fear inducing situation generalizes to other situations [7], [18], [48]. However, norBNI did not reduce the amount of freezing expressed in shock rats placed in a novel chamber. We also report the unexpected finding that the high dose of norBNI produced an increase in the freezing expressed by nonshock rats when re-exposed to the shock chamber. While speculative at this point, it is possible that the neural mechanisms that mediate freezing in shock rats may be different than the freezing expressed by nonshock rats. As such, the results of our experiments would suggest that KORs play a different role in innate fear mechanisms than it does in the fear associated with an intense stress experience.

It is well-documented that rodents pre-exposed to a short episode of relatively intense electrical shock show enhanced avoidance to novel situations and objects [8], [10], [14]–[17] which is believed to reflect behavioral responses associated with anxiety [46]. The present study used a similar protocol to examine the potential role of KORs in the lasting anxiety that can develop following a severe stressor. In the defensive withdrawal test, we show that the high dose of norBNI attenuated the latency for shock rats to leave the safety of the withdrawal box. Latency appears to be the most sensitive measure of anxiety in the defensive withdrawal test as shown in a previous study using a similar shock protocol to the one used in the present study [16]. In that study, the benzodiazepine anxiolytic drug decreased the latency to enter the open field in shock rats while having no effect on the time spent outside the withdrawal box or the number of entries in the open field. In the present study, there was non-significant tendency for norBNI to decrease latency in nonshock rats but norBNI's anxiolytic effects were specific for shock rats. We also found that norBNI decreased the time shock and nonshock rats spent in the withdrawal box. As previously shown and discussed [49], it is likely that the time spent in the withdrawal box represents a behavioral pattern that includes more than just anxiety because there is a clear dissociation in the rate and the extent in which latency and the time spent in the withdrawal box habituate over repeated daily tests. This indicates that the time spent in the withdrawal box is more than an avoidance behavior and that norBNI effects on this measure is not entirely dependent on the shock experience. Further work will be needed to completely characterize the role of KORs in the anxiety that is produced by exposure of rats to footshocks and to confirm the anxiolytic effect of KOR antagonists in this model of PTSD. Anxiolytic effects of norBNI were also noted in one study in non-stressed rats tested on the elevated plus maze [29]. As such, the results of this study using the defensive withdrawal test are consistent with studies indicating that KOR antagonists have anxiolytic effects in a variety of anxiety tests including the elevated plus maze, novelty-induced hypophagia and defensive burying protocols [29]–[30], [33], [50].

A number of inconsistencies were observed between the results of the fear expression and the defensive withdrawal tests. First, norBNI treated nonshock rats displayed an increase in freezing to the shock chamber while the same animals did not show an increase in avoidance in the defensive withdrawal test. Second, avoidance in the defensive withdrawal test was reduced by norBNI but generalized fear to a novel context was not affected. Since fear and anxiety represent different types of defensive behaviors, the apparent discrepant findings may be due to potential differences in KORs involvement in fear and anxiety. Fear is a defensive response associated with specific context or stimulus which has been associated with a negative emotional state (for example, re-exposure to the shock context) [51]–[53]. Freezing to the novel chamber following exposure of rodents to footshocks of similar intensity as used in the present experiments appears to result largely from a generalization of fear from the footshocks experienced in the shock box to other chambers [13]. In contrast, anxiety is conceptualized as behavioral response associated with a non-specific threat and is often operationally defined as an increase in avoidance behaviors [54]–[57]. The defensive withdrawal test is designed to measure the avoidance tendency of rodents that are naturally motivated to explore new environments [43] and the results of our experiments are consistent with previous studies which have consistently shown that norBNI and other KOR antagonists have anxiolytic effects using similar tests.

Contrary to what might be expected from the published literature on norBNI, it is somewhat surprising that norBNI given at a systemic dose of 15 mg/kg had no anxiolytic effect. For example, a number of studies have shown that systemic injections of norBNI given at 10 mg/kg had antidepressant and anxiolytic effects in mice and rats [29], [34], [37], [58]. However, other studies have reported that the KOR antagonists norBNI [59] and 5′-guanidinonaltrinodole or GTNI [26] given systemically at a dose of 10 mg/kg had no antidepressant effects in the swim test while the same studies showed that these KOR antagonists given as 20 µg in the cerebral ventricles had the predicted effect [26], [59]. Another recent study has reported that a 10 mg/kg systemic dose had no behavioral effects whereas a 30 mg/kg dose interfered with alcohol self-administration [60]. As discussed previously, a dose of 10–15 mg/kg should be sufficient to occupy KOR in body, but the binding of KOR in the brain following systemic injections of norBNI may be limited by bioavailability of norBNI to brain tissue [59]. For example, the more lipophilic KOR 5′acetamidinoethylnaltrindole or ANTI was found to have antidepressant effects following systemic dosing at 10 mg/kg whereas the less lipophilic GTNI did not [26]. Other potential reasons that may explain why the 15 mg/kg dose was ineffective in the present study include factors such as species type, age and weight of the subjects, type of behavioral test, time between the drug administration and the behavioral test, and presence of an intense stress episode. It is important to note that the anxiolytic effects of the 30 mg/kg dose of norBNI were from tests done 4 weeks after the drug treatment and that the design of our experiment does not rule out the possibility that the anxiolytic effects of norBNI might have been through some secondary mechanism.

It is difficult to compare the magnitude of the effects observed in our study to other studies because different studies have used different behavioral tests, KOR antagonists and experimental animals. However, in one study, rats given the 30 mg/kg dose of norBNI had open arm time that reached 30% which is typical for non-anxious rats tested on the elevated plus maze [29]. The anxiolytic effects reported here could be considered comparable to that study in that shock rats treated with the 30 mg/kg dose had latency responses that were similar to nonshock rats. One potential concern in the discussion of the mechanism mediating the anxiolytic effect of norBNI with the 30 mg/kg dose is that this concentration of the antagonist is approaching a concentration that would antagonize other opioid receptors (Endoh et al., 1992; Thomas et al., 2004). As such, one has to think about the possibility that norBNI produces its anxiolytic effect through non-KOR mediated mechanism. This is especially important considering that antagonism of other opioid receptors has also been shown to have anxiolytic properties [61]–[64]. However, this does not appear to be the case because antagonism of mu or delta opioid receptors lasts for a few hours after norBNI administration [39], [42] while the anxiolytic profile of norBNI lasts for weeks in the present study. Another caveat is that long-term inactivation of KOR may produce some compensatory changes in other neural mechanisms which in turn could be responsible for the anxiolytic effects of norBNI observed in the present study. This could represent an interesting area of investigation for future studies.

According to the present study, the KOR appears to be involved in avoidance in rats exposed to an acute episode of footshocks. More importantly, systemic injections of the KOR antagonist norBNI two days after the footshock episode were shown to block avoidance-like behaviors 4 weeks after the drug administration. These findings have potential implications for the treatment of humans with PTSD since fear associated with memories of the trauma often becomes generalized in a way that the person feels constantly threatened [65]–[67]. This can eventually lead to avoidance of many situations that may not be directly related to the trauma and the development of maladaptive behaviors including social isolation [3]. It is possible that treating PTSD patients with a KOR antagonist after a trauma experience may help reduce the development of the avoidance and anxiety that contribute to the psychological distress associated with the condition. In addition, the results presented here point to the potential of KOR antagonists as a form of prophylactic treatment in the development of symptoms of avoidance in at risk individuals experiencing a severe trauma.
